# Identification of candidate genes associated with bacterial and viral infections in wild boars hunted in Tuscany (Italy)

**DOI:** 10.1038/s41598-022-12353-8

**Published:** 2022-05-17

**Authors:** M. C. Fabbri, A. Crovetti, L. Tinacci, F. Bertelloni, A. Armani, M. Mazzei, F. Fratini, R. Bozzi, F. Cecchi

**Affiliations:** 1 Dipartimento di Scienze e Tecnologie Agrarie, Alimentari, Ambientali e Forestali, Università di Firenze, Firenze, Italy; 2 Dipartimento di Scienze Veterinarie, Università di Pisa, Pisa, Italy

**Keywords:** Genetic association study, Genetic markers, Genomics, Genotype, Population genetics, Infectious diseases, Bacterial infection, Viral infection

## Abstract

Wild boar (*Sus scrofa* L.) is one of the large mammals most spread worldwide, highly adaptable, and its population rapidly increased in many areas in Europe, including Italy, where Tuscany is considered particularly suitable for wild boar. Wild boars are potential hosts for different etiological agents, such as *Brucella* spp., *Leptospira* spp. and Pseudorabies virus and they can contribute to maintain and/or to disseminate some bacterial or viral pathogens to humans and domestic animals, above all-in free-range farms. In order to identify hypothetical genomic regions associated with these infection diseases, 96 samples of wild boars hunted in Tuscany during the 2018–2019 and 2019–2020 hunting seasons were considered. Diagnosis was achieved by serological tests and 42 Pseudorabies, 31 Leptospira and 15 Brucella positive animals were identified. All animals were genotyped with Geneseek Genomic Profiler Porcine HD (70 k) and a genome-wide scan was then performed. Significant markers were highlighted for Pseudorabies (two SNPs), Brucella (seven SNPs), and Leptospira (four SNPs) and they were located within, or nearby, 29 annotated genes on chromosome 6, 9, 12, 13, 14 and 18. Eight genes are implicated in viral (SEC14L1, JMJD6, SRSF2, TMPRSS2, MX1, MX2) or bacterial (COL8A1, SPIRE1) infections, seven genes (MFSD11, METTL23, CTTNBP2, BACE2, IMPA2, MPPE1 and GNAL) are involved in mental disorders and one gene (MGAT5B) is related to the Golgi complex. Results presented here provide interesting starting points for future research, validation studies and fine mapping of candidate genes involved in bacterial and viral infections in wild boar.

## Introduction

The wild boar (*Sus scrofa* L.) is widely distributed throughout Eurasia from Europe to the Far East, including SouthEast Asia, and extending to North Africa^1^; it is considered the second most abundant ungulate species in Europe^2^.

In Italy, the wild boar population is widely diffused^3^, reaching high-density levels in specific areas^4^ and Tuscany is particularly suited to the wild boar. This is evidenced by the high number of animals hunted in this area^3–5^.

The high density of wild boar in a particular area is a serious problem for the agricultural economy, causing extensive damage to croplands^6^ and may represents a severe hazard for both animals and human health.

In fact, it is known that wild boar can be the host for different etiological agents, thus contributing to maintaining and/or disseminating important zoonotic diseases^7^, as well as leptospirosis and brucellosis. Furthermore, other not-zoonotic diseases, such as the Pseudorabies (PrV) or Aujeszky’s disease, also spread by wild boar have a large economic impact on the swine industry.

Brucella is a zoonotic Gram-negative bacterium. Among the different species, *B. abortus*, *B. suis*, and rarely, *B. melitensis* can infect swine as well as wild boar (*Sus scrofa*)^8–10^. Tuscany, as many other Italian regions, is free from bovine and ovine brucellosis from several years, thanks to the progress of the eradication plan implemented throughout the country since 1992 and 1994 (D.M. 2/7/92 n. 453; D.M. 27/8/94 n. 651; EFSA 2017). *B. suis* biovars 1 and 3 are rarely reported in Europe, while *B. suis* biovar 2 (bv. 2) is largely diffused in East Europe, and it was recently introduced in Italy where it was isolated from domestic pigs and wild boar^8,10,11^. Wild boar represents one of the main reservoirs of *B. suis* bv. 2, which is responsible for reproductive disorders such as infertility, abortion, stillbirths, decreased litter size, weak piglets, orchitis and epididymitis in males, and focal abscess formation^10^. Recently, *B. suis* bv. 2 has been detected also in cows, in which seroconversion was detected without the presence of clinical signs^12,13^. Human infections by this serovar are rarely reported^11^.

Leptospirosis represents a re-emerging worldwide zoonotic disease; it is caused by *Leptospira* spp., a Gram-negative spirochetal bacterium. The genus *Leptospira* is traditionally divided into more than 260 antigenically-different serovars, at present classified as pathogenic, intermediate, and saprophytic, with different levels of pathogenicity for animals and humans^14,15^. Swine are the reservoir host for some serovars (Pomona, Tarassovi, and Bratislava), but it is not excluded that it could be infected by many others serovars^16^. Wild boars, due to their natural behaviour and in relation to the geographical area where they live, are often infected by several serovars such as Icterohaemorrhagiae, Grippotyphosa and Canicola^17–19^. As demonstrated by several studies, Central Italy, and in particular Tuscany, present some environmental and geographic features that promote the *Leptospira* spread. Wild boar and feral pig live in contact with domestic animals and humans, representing one of the most important *Leptospira* reservoir among wildlife. For this reason, wild boar is considered an “indicator” of leptospirosis in those area where many different species are forced to co-exist^20–22^.

Pseudorabies or Aujeszky’s disease (PrV) is caused by Suid herpesvirus 1 which belongs to the Herpesviridae family, subfamily Alphaherpesvirinae, genus Varicellovirus^23^. This virus, as the other members of the afore mentioned family, is enveloped with a double-stranded DNA genome. Although domestic swine and wild boar represent the natural host of PrV, the virus can also infect numerous wild and domestic mammals including ruminants, carnivores, and rodents^24,25^.

Pseudorabies virus circulates in domestic swine and wild boar (*Sus scrofa*) populations in several countries^26^. The PrV wild boar seroprevalence in Europe ranges from 4 to 66%, representing a risk of infection for domestic swine and other susceptible animals^26–29^. Consequently, the role of wild boars in the epidemiology of the Pseudorabies is of primary importance, because it represents a serious threat to the completion of the European Community eradication program.

Further knowledge on alternative methods to control the spread of the disease should therefore be investigated. The use of disease-resistant livestock breeds that reduce infection pressure with a decreased incidence of disease is therefore of considerable importance^30^.

Virulence of pathogens and susceptibility of wildlife species to disease are influenced by attributes of the host and the pathogen. Discovering the factors that determine host susceptibility/resistance to disease contributes to prevent diseases in both human and domestic animals and improve “global health”. A wide range of genetic variations in disease resistance has been observed in swine regarding different viral, bacterial, and parasitic diseases^30^.

Previous studies have reported that genetic diversity of individuals and populations of wildlife, at both neutral loci and functional genes, is related to disease tolerance or resistance. Indeed, high levels of inbreeding^31,32^ with a consequent heterozygosity reduction at neutral microsatellite markers can increase susceptibility to disease in wildlife^33–35^. Acevedo-Whitehouse et al.^36^ highlighted that a higher genetic heterozygosity was associated with lower probability of infection of bovine tuberculosis in wild boar.

The major histocompatibility complex (MHC), a highly polymorphic family of vertebrate genes involved in initiation and regulation of the immune response, has been the target of considerable investigation of disease resistance and tolerance in wildlife^37,38^. Recent advances in genome sequencing, and particularly in the development of high-density single nucleotide polymorphism (SNPs) arrays, have improved genome-wide screening and, therefore, our ability to detect disease-associated genes^39^ and to identify the genetic control of disease resilience^40^. For example, in wild boar**,** Queirós et al.^39^ found candidate genes (i.e. LOC102164072, BDNF/NT-3, NTRK2, CDH8, IGSF21) for host genetic susceptibility to tuberculosis.

Given the importance of these pathogens in animals and considering the potential risk factor for human diseases, our study is one of the first attempts to identify genomic regions associated with infections from Pseudorabies virus, Brucella spp. and Leptospira spp. in wild boar.

## Results

Table 1 reports the prevalence of positive samples over the total of 96 for Pseudorabies, Brucella and Leptospira. Positivity to two etiological agents was detected only in 5/96 samples (5.21%; 95% CI 2.00–8.42%). Eight were the negative samples. To calculate the confidence interval (CI) and to assess the prevalence, a binomial logistic regression was performed.

**Table 1:** Number of positive animals and prevalence of Pseudorabies, Brucella and Leptospira in the 96 animals collected from Tuscany area.

Infection	Total analysed animals	Positive animals	Prevalence (%)	95% CI
Pseudorabies	96	42	48.9	36.58–50.92
Brucella	96	15	15.6	10.28–20.37
Leptospira	96	31	32.3	25.54–39.05

Genome-wide scan was performed, and 13 significant SNPs were identified, as shown in Table 2.

**Table 2:** List of significant SNPs identified for each disease.

Infection		Name	Chr	Position	Pvalue	Type of Variant	Within	Upstream	Downstream
Pseudorabies	ASGA0084173	rs81341734	12	4,612,583	4.12E−05	Intergenic variant	–	*SEC14L1, MGAT5B*	*JMJD6, MXRA7, MFSD11, METTL23, SRSF2*
	WU_10.2_18_30218795	rs339406967	18	28,399,958	3.04E−05	Intron variant	*CTTNBP2*	–	*CFTR*
Brucella	MARC0040908	rs81232015	9	58,693,183	1.55E−05	Intron variant	*NTM*	–	–
	MARC0029225	rs81224033	9	58,592,360	1.79E−05	Intron variant	*NTM*	–	–
	ALGA0073505	rs80987534	13	190,711,065	1.86E−05	Intergenic variant	–	–	–
	WU_10.2_13_200912860	rs319979903	13	190,770,452	1.86E−05	Intergenic variant	–	–	–
	ASGA0060211	rs81443008	13	204,883,958	2.44E−05	Intron variant	*TMPRSS2*	*BACE2, FAM3B, MX1, MX2*	*RIPK4*
	MARC0024545	rs80808271	13	159,240,192	4.78E−05	Intron variant	*COL8A1*	*CMSS1, FILIP1L*	–
	ALGA0072626	rs80896010	13	159,213,442	4.78E−05	Downstream gene variant	–	*CMSS1, FILIP1L*	*COL8A1*
Leptospira	H3GA0053117	rs81338390	6	97,242,874	1.12E−05	Upstream gene variant	–	*SPIRE1, PRELID3A, AFG3L2, TUBB6*	*CIDEA, IMPA2, MPPE1, CHMP1B, GNAL, TRNAG- UCC*
	WU 10.2 13 10,381,840	rs324503493	13	9,111,717	2.05E−05	Upstream gene variant	*ZNF385D*	–	–
	H3GA0042130	rs80982803	14	114,457,304	1.99E−05	Intron variant	*NEURL1*	*INA, PCGF6, TAF5, MIR1307, USMG5, PDCD11, CALHM3*	*SH3PXD2A*
	ASGA0066225	rs80944071	14	114,471,571	1.99E−05	Intron variant	*NEURL1*	*INA, PCGF6, TAF5, MIR1307, USMG5, PDCD11, CALHM3*	*SH3PXD2A*

The SNP name, the chromosome and pb positions, p-value, type of variant and the list of the candidate genes in the regions flanking the significant markers (± 250,000 nucleotides) are reported.

**Figure 1:**
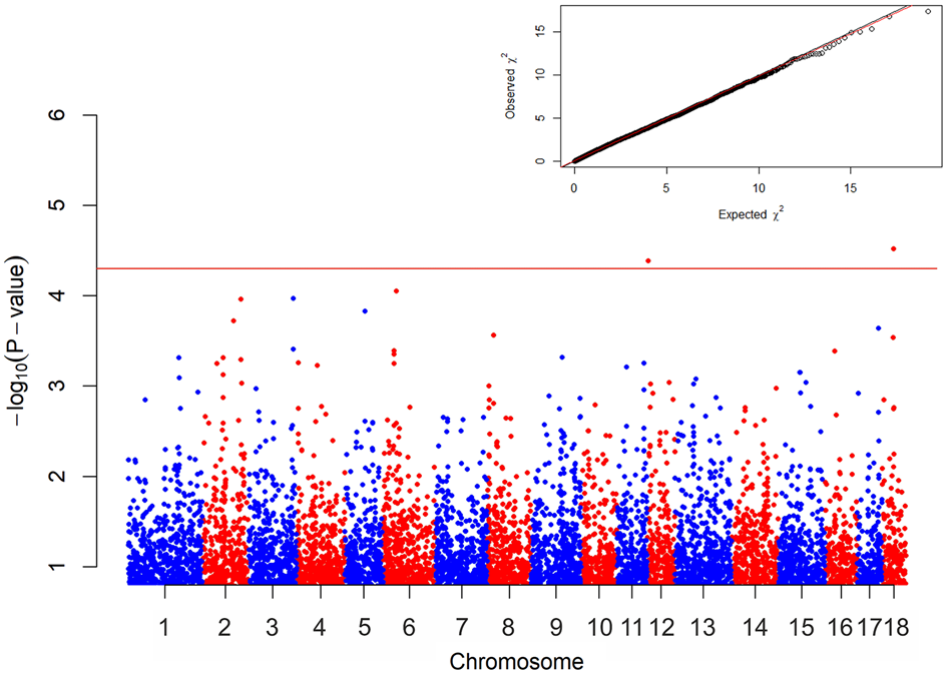
Manhattan plot of the test values obtained, for each marker for Pseudorabies virus. The *horizontal red line* separated the two most significant markers (P < 0.00005).

Two SNPs were significantly associated to Pseudorabies (Fig. 1).

ASGA0084173 is situated on chromosome 12, with *SEC14L1* and *MGAT5B* genes upstream located and *JMJD6*, *MXRA7*, *MFSD11*, *METTL23*, *SRSF2* downstream located; WU_10.2_18_30218795 is on chromosome 18, within *CTTNBP2* gene and upstream to *CFTR* gene.

**Figure 2:**
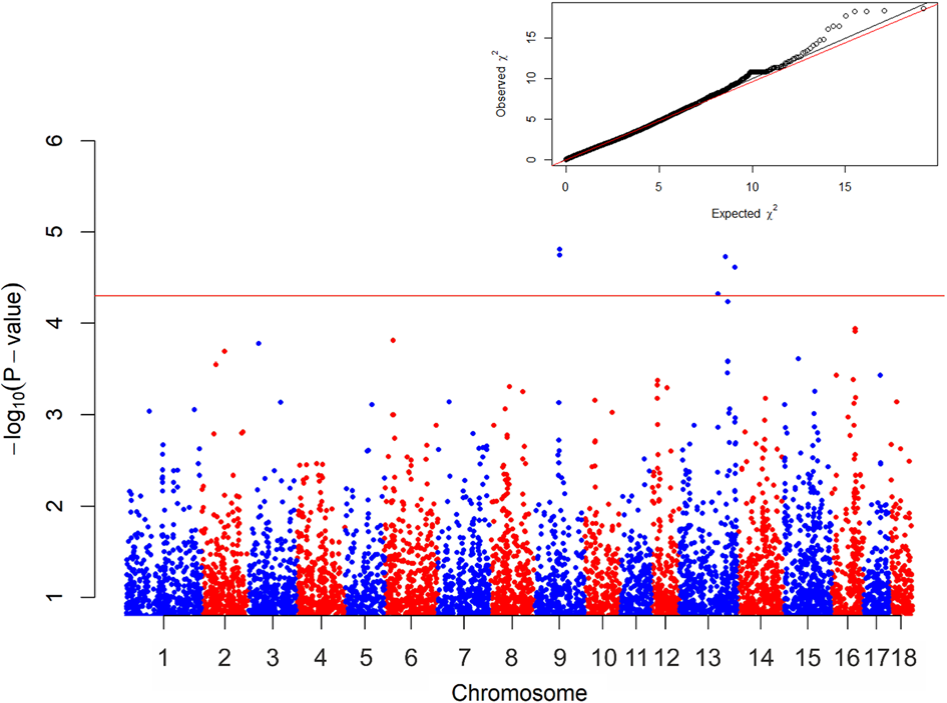
Manhattan plot of the test values obtained, for each marker for Brucella. The *horizontal red line* separated the seven most significant markers (P < 0.00005). ALGA0073505, WU 10.2 13 200912860, MARC0024545 and ALGA0072626 on Chromosome 13 have an extremely closed position, difficult to separate graphically.

Several SNPs were identified analysing Brucella infection (Fig. 2), precisely, seven SNPs are found significantly associated with this disease.

Both MARC0040908 and MARC0029225 markers are on chromosome 9 within *NTM gene*. 250 Kbp upstream and 250 Kbp downstream of the aforementioned SNPs no genes are found (Table 2).

The other five significant SNPs are located on chromosome 13: two of them, namely, MARC0024545 and ALGA0072626, are situated in a close region, sharing *CMSS1* and *FILIP1L* genes, which are found upstream to the two SNPs. MARC0024545 is an intron variant because within *COL8A1*, while ALGA0072626 is upstream to the aforementioned gene. ALGA0073505 and WU_10.2_13_200912860 markers are in a genomic region where no characterized genes are present. ASGA0060211 is classified from VeP database as intron variant because it is positioned within *TMPRSS2* gene. *BACE2*, *FAM3B*, *MX1* and *MX2* genes are upstream and *RIPK4* is downstream to the SNPs.

**Figure 3:**
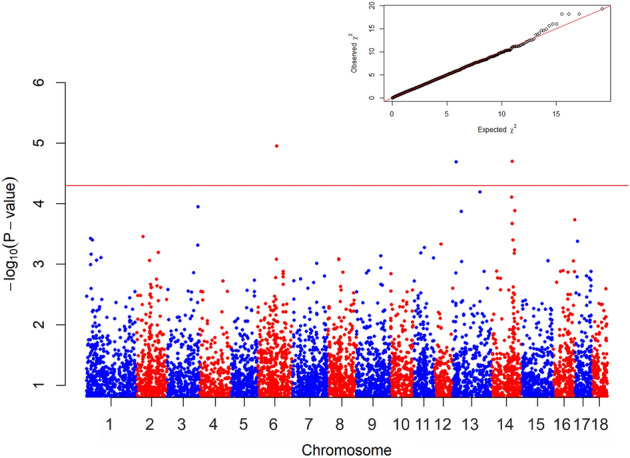
Manhattan plot of the test values obtained for Leptospira. The *horizontal red line* separated the four most significant markers (P < 0.00005). SNP H3GA0042130 and ASGA0066225 on Chromosome 14 have an extremely closed positions, difficult to separate graphically.

Figure 3 described the four significant associated SNPs for Leptospira.

The H3GA005311 is located on chromosome 6, in a genomic window rich of genes: *SPIRE1*, *PRELID3A*, *AFG3L2* and *TUBB6* are upstream and *CIDEA*, *IMPA2*, *MPPE1*, *CHMP1B*, *GNAL* and *TRNAG-UCC* are downstream of this marker. WU_10.2_13_10381840 has been found within *ZNF385D* gene. H3GA0042130 and ASGA0066225 are close to each other with less than 15 Kbp between the two SNPs. Both are located on *NEURL1* gene, with *INA*, *PCGF6*, *TAF5*, *MIR1307*, *USMG5*, *PDCD11*, *CALHM3* upstream and *SH3PXD2A* downstream genes.

**Figure 4:**
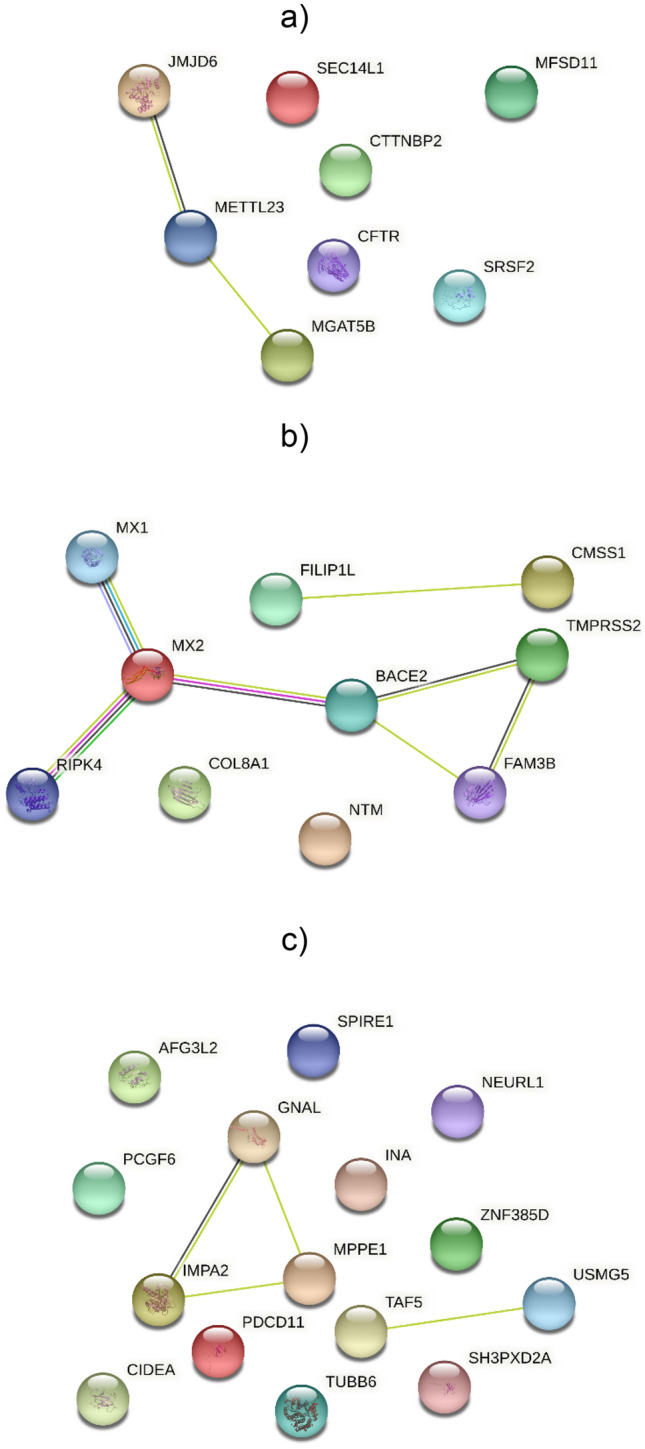
Protein networks of GWAS genes according to STRING database. (**a**) Pseudorabies; (**b**) Brucella; (**c**) Leptospira. Nodes are proteins; lines indicate interactions between proteins with: pink lines for known interactions experimentally determined, sea blue for interactions derived by curated databases. For the predicted interactions, green is for neighbourhood gene, red lines for gene fusions and blue lines for gene co-occurrence. Black lines are for co-expression, light green lines for text mining and light blue lines for protein homology. Protein interactions include direct (physical) and indirect (functional) associations derived from different sources (genomic context, high through-put experiments, conserved co-expression, previous knowledge).

STRING software was used to analyse the possible interactions among proteins encoded by identified genes. Figure 4 showed the results for each infection.

Three of the nine genes (*JMJD6*, *MGAT5B* and *METTL23*, Fig. 4a) for Pseudorabies infection are linked. Brucella phenotype showed the greater interactions among genes: two clusters are identified, the first between *FILIP1L* and *CMSS1*, and the second one among *MX1*, *MX2*, *RIPK4*, *TMPRSS2*, *BACE2* and *FAM3B* genes (Fig. 4b). Although Leptospira was the infection with more genes found in the significant genomic window, few genes are linked to each other (*IMPA2*, *MPPE1*, and *GNAL*—*TAF5* and *USMG5*, Fig. 4c).

Using the PANTHER software, it was possible to summarize the biological processes and molecular functions in which the identified candidate genes are involved (Table 3). For all the infections, the functional genes were enriched in “GO: 0065007, biological regulation”, “GO: 0009987, cellular process”, “GO: 0051179, localization” and “GO: 0008152, metabolic process”. Furthermore, the functional genes of Leptospira were enriched in many other processes including “GO: 0000003, reproduction” and “GO: 0022414, reproductive process”. More molecular functions were detected for genes involved in Leptospira infection than for Pseudorabies and Brucella infections.

**Table 3:** Biological process and molecular function Gene Ontology (GO) terms enrichment analysis results.

Infection	Biological process GO terms	Molecular function GO terms
Pseudorabies	GO: 0065007, biological regulation; GO: 0009987, cellular process; GO: 0051179, localization; GO: 0008152, metabolic process	GO: 000548, binding; GO: 0003824, catalytic activity; GO: 0005215, transporter activity
Brucella	GO: 0065007, biological regulation; GO: 0009987, cellular process; GO: 0051179, localization; GO: 0008152, metabolic process	GO: 000548, binding; GO: 0003824, catalytic activity; GO: 0005215, transporter activity
Leptospira	GO: 0065007, biological regulation; GO: 0009987, cellular process; GO: 0051179, localization; GO: 0008152, metabolic process; GO: 0032502, developmental process; GO: 0051704, multi-organism process; GO: 0032501, multicellular organismal process; GO: 0000003, reproduction; GO: 0022414, reproductive process; GO: 0050896; response to stimulus; GO: 0023052, signalling	GO: 000548, binding; GO: 0003824, catalytic activity; GO: 0005215, transporter activity; GO: 0005198, structural molecule activity; GO: 0098772, molecular function regulator

## Discussion

The seroprevalence estimate obtained for the three infections were higher than those observed in previous studies. In particular, the presence of PrV (48.9%) in our population is higher than what reported in the same area by Bertelloni et al.^41^ (28.6%) and in previous studies conducted in North-West Italy, indicating the importance of this animal species in the endemicity of Aujeszky’s disease^27^. The seroprevalence of PrV in wild boar has been already described in many European countries (France, Germany, Spain, Slovenia, Croatia, and Czech Republic) ranging from 4 to 66%^26,28,42–46^.

This investigation, point out also a higher seroprevalence of Brucella than that reported in other studies carried out in past years in Italy (0.00%^47^; 0.53%^41^, as well as for Leptospira in Italy (6.0%^47^; 15.3%^48^; 9.5%, 8.8%^41^) and in Europe (3.1%^17^; 10.4%^19^).

The results of the GWAS suggest that some genes might have a role in the resistance/susceptibility to the studied bacterial and viral diseases in wild boar, but no gene was found common to the three infections.

The genomic window 250 Kbp downstream and upstream to the significant SNPs contained a variable number of genes, ranged from 9 (Pseudorabies) to 20 (Leptospira), as reported in Table 2. Interesting is that several SNPs are close to each other, suggesting being in linkage disequilibrium and probably fixed. Two pairs of SNPs were found very close in Brucella infection, ALGA0073505—WU_10.2_13_200912860 (~ 182 Kbp of distance) and MARC0024545—ALGA0072626 (~ 26 Kbp of distance). The closest SNPs are H3GA0042130 and ASGA0066225, identified in Leptospira analysis, distant less than 15 Kbp.

Seven genes associated with Pseudorabies and involved in multiple biological processes are located on chromosome 12. For instance, the *MGAT5B* gene (alpha-1,6-mannosylglycoprotein 6-beta-N-acetylglucosaminyltransferase B) is related to the Golgi apparatus operating at the intersection of the secretory, lysosomal, and endocytic pathways. Several studies^49,50^ indicated that viral PrV envelopment and tegumentation occurs at late Golgi or post-Golgi compartments, suggesting that this gene may have a role in PrV virulence and dissemination. As reported in Fig. 4a, this gene is linked to other two genes of interest, the *JMJD6* (JmjC domain-containing protein) and the *METTL23* (methyltransferase like 23)**.** The *JMJD6* gene has many functions, ranging from a cell surface receptor for recognition of apoptotic cells to a nuclear factor responsible for lysine hydroxylation and arginine demethylation^51^. Recent reports indicating a multifactorial role in foot-and-mouth disease virus (FMDV) infection^52^, in tumorigenesis and virological interactions^53^. *METTL23* belongs to a family of methyltransferase like proteins (*METTL*) that transfer methyl group to various substrates and it is involved in human intellectual disability^54^. The same functionality has been attributed to the *MFSD11* (major facilitator superfamily domain containing 11) gene^55^.

On the same chromosome *SEC14L1* gene was interestingly detected (SEC14 like lipid binding, also called *PRELID4A*) and it interacts with RIG-I, a cytosolic pattern recognition receptor, which has found to be required for the activation of anti-PrV activity^56^.

*MXRA7* (matrix remodelling associated 7) is highly expressed in murine and human ocular tissues and might play a role in pathological processes or diseases involving injury, neovascularization and wound healing^57^. Another study^58^ reports evidence for a protective role in the mouse psoriatic epidermis. Moreover, *MXRA7* gene might be involved in bone marrow mesenchymal stem cells (BMSCs) functions^59^.

*SRSF2* (serine and arginine rich splicing factor 2; also known as SFRS2) gene plays vital roles in a number of biological and pathological processes and it is associated in humans with the progression of a variety of diseases, including viral infection and tumorigenesis^60^.

On chromosome 18, *CFTR* (Cystic fibrosis transmembrane conductance regulator) and *CTTNBP2* (cortactin binding protein 2) genes were detected. It is interesting the role of CTTNBP2 in cattle and humans. In Brown Swiss cattle breed this gene is involved in a recessive neurological disease^61^, i.e. the Bovine Progressive Degenerative Myeloencephalopathy (Weaver Syndrome). It is considered as a good candidate gene in humans for a role in the pathogenesis of mental retardation^62^ such as autism-like behaviours^63^.

Genes linked to nervous system are probably expected to be found, because neurological signs predominate with Pseudorabies disease progression, e.g. ataxia, circling, paresis and paralysis^64^.

Several genes associated with host resistance or susceptibility to Brucella spp. have been identified in cattle^65^, buffalo^66^, goats^67,68^, humans and pigs^69^, from those different type of Collagenases were found. In this research the Collagen type VIII Alpha 1 chain (*COL8A1)* is identified*. COL28A1* has been previously associated with antibody response in feral swine (Sus scrofa) infected with Brucella suis^69^, suggesting that *COL8A1* might play an analogue role in wild board.

Genes implicated in viral infections are *MX1* (Interferon-induced GTP-binding protein Mx1) and *MX2* (Interferon-induced GTP-binding protein Mx2)^70^. Mx proteins are interferon (IFN)-induced dynamin-like GTPases that are present in all vertebrates and are known to inhibit the multiplication of several viruses^71^, including vesicular stomatitis virus (VSV)^72^, influenza A virus (FLUAV)^73^ and classical swine fever virus (CSFV)^74–76^. Furthermore, recent studies have shown that MX1 inhibits the replication of foot-and-mouth disease virus (FMDV) (as *JMJD6* found associated with Pseudorabies), and bovine viral diarrhoea virus (BVDV)^77^. Finally, porcine *MX2* was also found to have the antiviral activity against Porcine reproductive and respiratory syndrome virus (PRRSV)^78^.

As STRING software highlighted, the Mx genes are flanked by *BACE2*, *TMPRSS2*, *FAM3B* and *RIPKA* genes. The *TMPRSS2* (transmembrane serine protease 2) gene encodes a serine protease that can process the influenza A virus hemagglutinin into its fusion-competent state in human airway epithelial cells^79^ and it is mainly involved in SARS-CoV and SARS-CoV-2 infections^80^.

The second cluster that STRING identified for Brucella, was formed by *CMSS1* and *FILIP1L* genes, but no studies describing these two genes were found.

*NTM* (Neurotrimin) gene encodes a member of the IgLON (*LAMP*, *OBCAM*, *NTM*) family of immunoglobulin (Ig) domain-containing glycosylphosphatidylinositol (*GPI*)-anchored cell adhesion molecules. A study performed in humans suggested that *NTM* gene is associated with the level of the intelligence quotient (IQ) and genome wide association studies identified an association between *NTM* variation and cognitive function performances in humans^81,82^.

Among the genes associated with Leptospira disease here highlighted, several of them are previously found to be associated with human neurological disorders: *IMPA2*, *GNAL*, *MPPE1* and *AFG3L2. IMPA2* (CIDE-N domain-containing protein) gene has been associated with bipolar disorder, schizophrenia^83^ and febrile seizure^73^. *GNAL* (G protein subunit alpha L) gene has been linked to bipolar disorder and schizophrenia^84^. *MPPE1* (Metallophosphoesterase 1) clustered with *IMPA2* and *GNAL* genes through STRING software (Fig. 4c). Dysregulation of protein phosphorylation and subsequent abnormal cellular signalling has been postulated to be involved in neuropsychiatric disorders, thus making *MPPE1* a plausible biological candidate gene for bipolar disorder (BPD)^85^. *AFG3L2* (AFG3 Like Matrix AAA Peptidase Subunit 2) gene is a candidate gene for hereditary spastic paraplegias or neurodegenerative disorders (https://www.genecards.org/).

These results are interesting because human patients affected from Leptospira reported neurological manifestations in only 10–15% of cases^86^. The associations here identified suggest that nervous system could be more involved in wild boar Leptospira infections than in humans.

Another interesting gene is *SPIRE1* (KIND domain-containing protein) which belongs to the SPIRE family that emerged as a class of host cell factors that may affect the invasion process. Interestingly, *SPIRE1* has been implicated in the infection of *Salmonella typhimurium*^87^.

Other two genes of interest are the *PRELID3A* (PRELI/MSF1 domain-containing protein, also called *SLMO1*) and *TUBB6* (Tubulin Beta 6 Class V). In particular, the PRELI-like family proteins acted as lipid transporters and play an important role of embryonic and development lymphocyte differentiation. The PRELI-like family proteins have been proposed to involve many cellular functions including apoptosis, cellular lipid metabolism and cellular signalling and were correlated with several types of diseases and malignant tumours^88^. *TUBB6* gene was found associated with muscle differentiation and regeneration^89^.

In mouse, *CIDEA* (Cell Death Inducing DFFA Like Effector A) gene regulates thermogenesis, lipolysis, and conservation of energy and it is considered to be a proapoptotic factor^90^. The *SH3PXD2A* gene was studied in mouse and humans defining it as a potential risk gene for orofacial clefting, indeed, Cejudo-Martin et al.^91^ argued that disruption of the mouse *SH3PXD2A* gene was associated with complete cleft of the secondary palate in 50–90% of mutant mice.

*CALHM3* (Calcium Homeostasis Modulator 3) gene together with *CALHM1*, form a complex to mediate rapid taste neurotransmission; indeed, genetic deletion of *CALHM3* abolished sweet, bitter, and human taste perception^92^. Also, *NEURL1* (neuralized E3 ubiquitin protein ligase 1) gene has many functions, in particularly it was related to the cellular process involved in reproduction in multicellular organism^93^. Moreover, *NEURL1* has been associated with fat content in Nordic cattle breeds^94^, while in humans this gene was related to survival in Oesophageal adenocarcinoma (EAC) patients^70^.

On the same chromosome *PCGF6* (polycomb group ring finger 6) was found, which plays an essential role in embryonic development of mice and in mouse fertility^95^.

In summary, significant SNPs were detected to be associated with viral and bacterial disease. Furthermore, among the 29 genes highlighted, 18 genes could be considered candidate genes for genetic resistance or susceptibility to diseases. Indeed, identified genes are implicated in viral (*SEC14L1*, *JMJD6*, *SRSF2*, *TMPRSS2*, *MX1*, *MX2*) bacterial (*COL8A1*, *SPIRE1*), and neurological disorders (*MFSD11*, *METTL23*, *CTTNBP2*, *BACE2*, *IMPA2*, *MPPE1* and *GNAL*), or in the functions of the Golgi complex (*MGAT5B*), organelle where viral envelope is occurred. No candidate genes related to reproduction system were identified for Leptospirosis and Brucellosis, but it could be hypothesized that wild boar responses are slightly different from those reported on reared pigs and only with a greater sample size it would be possible to individuate the association. In addition, the interesting findings of genes not directly related to infection symptoms, are intriguing, suggesting that further studies are needed to better clarify the pathways of these diseases. Results presented here represent interesting areas for future research, validation studies and fine mapping of candidate genes involved in bacterial and viral infections in wild boar.

## Methods

### Statement of animal rights

The wild boars were not hunted for the purpose of this study and none of the authors were involved with the hunting. Animals were hunted following regional hunting laws (Regolamento di attuazione della legge regionale 12 gennaio 1994 n 3 DPGR 48/R/2017). Thus, in accordance to the 2010/63/EU guide and the adoption of the Law D.L. 04/03/2014, n.26 by the Italian Government, an ethical approval was not required for this study.

### Sample collection

96 wild boars (54 females and 42 males) hunted in Tuscany during the 2018–2019 and 2019–2020 hunting seasons (from November to January) were sampled. The study was part of the project PRA_2018_56 financed by University of Pisa and entitled “Evaluation of hygienic-sanitary and qualitative parameters of wild boars hunted in Tuscany and Liguria”^14,29,41^, which had the purpose to investigate the role of the wild boar in the epidemiology of some infectious diseases for livestock and humans.

Animals were hunted in different areas in the provinces of Pisa (34, from 5 different localities), Siena (20, from 2 localities), Grosseto (35, from 11 localities), and Livorno (7, from 5 localities), characterised by the abundant presence of wild boars and other wild ungulates^96^. At postmortem examination, samples did not present relevant lesions related to infectious disease. During necroscopy, the kidneys were collected, and serum was extracted from the infraorbital cavities^97^.

### Serological analysis for Pseudorabies, Brucella and Leptospira infections

Serum samples were analysed by ID Screen Aujeszky gB competitive kit detecting anti-gB PrV antibodies (ID.vet, Grabels, France). Test procedures and interpretation of results were performed according to the manufacturer’s instructions, adopting the short serum incubation protocol. The optical density was measured by a plate reader (Multiscan FC; Thermo Scientific, Waltham, MA, USA) at 450 nm wave-length.

Rose Bengal Test (RBT) and complement fixation test (CFT) were employed to detect anti-brucella antibodies. RBT and CFT were performed as described by World Organization for Animal Health (OIE) (OIE 2016); antigens used in both tests were obtained from the ‘‘Istituto Zooprofilattico Sperimentale dell’Abruzzo e del Molise G. Caporale, Teramo”.

Leptospira antibodies were detected by microscopic agglutination test (MAT) as previously described^11^, titers of 1:100 were considered positive. The serovars were employed as live antigens in MAT (Supplementary Information). Anti-serum has been used as positive control for each investigated serogroup provided by ‘‘Istituto Zooprofilattico Sperimentale della Lombardia ed Emilia Romagna, Brescia’’, while sterilized saline water was used as a negative control.

### DNA extraction and SNPs quality control (QC)

Ninety-six wild boars were genotyped using the “Geneseek Genomic Profiler Porcine HD (70 k)”, containing 62,330 SNPs. SNP genotyping was outsourced at the Science and Technology Park of Sardinia (Porto Conte Ricerche; https://www.portocontericerche.it/it).

Total DNA extraction for each sample of kidney was performed, starting from about 100 µl of homogenised tissue, according to the salting out procedure proposed by Armani et al.^98^, further modified and applied as Salting out reference protocol by Armani et al.^99^. Final DNA concentration and purity were assessed with Nanodrop ND-1000 spectrophotometer (NanoDrop Technologies, Wilmington, DE, US) by two subsequent measurements of the absorbance value at 260 nm and calculation of A260/A280 and of A260/230 ratios. 260/280 and 260/230 values ≥ 2 were considered indicative of nucleic acid purity according to the manufacturer’s indications (https://tools.thermofisher.com/content/sfs/brochures/TN52646-E-0215M-NucleicAcid.pdf). DNA samples were prepared in a volume of 30 ul per concentration of 50 ng/ul in 96 plates and sent to the laboratory for genotyping.

The SNPs quality control (QC) has performed with PLINK v.1.07 (http://zzz.bwh.harvard.edu/plink/) and only autosomal SNPs with a call rate higher than 95%, a minor allele frequency (MAF) > 1% and with no extreme deviation from Hardy–Weinberg equilibrium (P value > 0.00001) were included in the analysis. Animals with more than 5% of missing genotypes were discarded. After QC, 42,431 SNPs mapped on the 18 porcine autosomes and 93 individuals were retained. The number of SNPs per chromosome is reported in Table 4.

**Table 4:** Total number of SNPs before quality control (pre-QC), post quality control (post-QC) and the percentage of SNPs retained for each autosomal chromosome.

Chromosome	Total markers pre-QC	Total markers post-QC	%Markers post-QC
1	5926	4054	9.55
2	4141	2546	6.00
3	3575	2600	6.13
4	3807	2626	6.19
5	3009	2103	4.95
6	4360	2870	6.76
7	3943	2823	6.66
8	3533	2376	5.60
9	3856	2730	6.43
10	2873	1967	4.63
11	2344	1623	3.82
12	2513	1526	3.59
13	4435	3088	7.28
14	4087	2556	6.02
15	3607	2534	5.97
16	2386	1748	4.12
17	2178	1350	3.18
18	1757	1311	3.09

### Genome wide association study and gene set enrichment analysis

The analysis was carried out for each infection separately, evaluating healthy vs infected animals. The association analysis was carried out with GenABEL^100^, which performs a simple linear regression marker-phenotype analysis. Firstly, the genomic relationship matrix was calculated with the function *ibs* (https://rdrr.io/cran/GenABEL/man/ibs.html), where for a given pair of individuals i and j, the identical by state coefficients (f_i, j_) is calculated as follows:

$${f}_{i,j}= \frac{1}{N}\sum k \frac{\left({x}_{i,k}- {p}_{k}\right)\left({x}_{i,k}- {p}_{k}\right) }{{p}_{k}(1- {p}_{k})},$$

where *N* is the number of markers used, *x*_i, k_ is the genotype of the ith individual at the kth SNP (coded as 0, ½ and 1), *p*_k_ is the frequency of the reference allele and k = 1,…, N.

Then, the additive polygenic model described below was applied:

$$Y=X\beta +a+e.$$

Each phenotype has been analysed separately (affected: Pseudorabies 42; Leptospira 31; Brucella 15); β was a vector with the fixed sex effect and **X** was the incidence matrix that associated each observation to levels of factor in β. The random effects in the model were the animal and the residual, which were assumed normally distributed as α∼N $\left(0, G{\upsigma }_{g}^{2}\right)$ and e∼N $\left(0, I{\upsigma }_{e}^{2}\right)$, where **G** was the genomic relationship matrix, **I** is an identity matrix, and ${\upsigma }_{g}^{2}$ and ${\upsigma }_{e}^{2}$ are the additive genomic and residual variances, respectively. Regression was performed using the GenABEL function *mmscore* and the associations between marker and phenotype with a *P* value ≤ 5 × 10^–5^ were considered significant^101^. The aforementioned threshold was used considering that the study was carried out on wild boar, which has not a species-specific SNP chip. Moreover, this level of significance for association is called “suggestive”, and it was introduced by Lander e Kruglyak^101^ and it is widely used in GWAS^102–105^. For each trait, a Manhattan plot and a quantile–quantile (Q–Q) plot were produced using the R software^103^ uploading chromosome position from *Sus Scrofa 10.2 assembly* (https://may2017.archive.ensembl.org/Sus_scrofa/Info/Index) to the recent *Sus* Scrofa 11.1 (https://www.ensembl.org/Sus_scrofa/Info/Annotation).

VeP database (https://www.ensembl.org/info/docs/tools/vep/index.html) was used to investigate the type of significant SNPs. A genomic window of 250 Kbp upstream and downstream from the significant SNP for each trait was investigated using the R package biomaRt^106,107^, which accesses the data available in Ensembl database (https://www.ensembl.org). The genes identification was based on Scrofa 11.1 assembly (https://www.ensembl.org/Sus_scrofa/Info/Annotation). For the Gene set enrichment analysis, the lists of protein coding genes were uploaded to STRING 11.5^108^ and PANTHER v.16.0^109^.

## Supplementary Information

Supplementary Information.

## Data Availability

The datasets analyzed during the current study are available online on the following link: https://osf.io/bz89n/.
