# Supplementary material for: Identification of candidate genes associated with bacterial and viral infections in wild boars hunted in Tuscany (Italy)
Source: Sci Rep. 2022 May 17;12:8145. doi: 10.1038/s41598-022-12353-8 (PMC9114367; doi:10.1038/s41598-022-12353-8)
Supplement: Supplementary file 1 — Supplementary Information. [file 41598_2022_12353_MOESM1_ESM.docx]

**Identification of candidate genes associated with Bacterial and Viral infections in Wild Boars hunted in Tuscany (Italy)**

# Fabbri M.C.^1^*, Crovetti A.^1^, Tinacci L.^2^, Bertelloni F. ^2^, Armani A. ^2^, Mazzei M. ^2^, Fratini F. ^2^, Bozzi R.^1^, Cecchi F. ^2^

**Supplementary Information.**

The serovars tested: Icterohaemorrhagiae (serogroup Icterohaemorrhagiae, strain Bianchi), Canicola (serogroup Canicola, strain Alarik), Pomona (serogroup Pomona, strain Mezzano), Grippotyphosa (serogroup Grippotyphosa, strain Moskva V), Tarassovi (serogroup Tarassovi, strain Mitis Johnson), Bratislava (serogroup Australis, strain Riccio 2), Hardjo (serogroup Sejroe, serovar Hardjoprajitno), Castellonis (serogroup Ballum, strain Castellon 3), Copenhageni (serogroup Icterohaemorrhagiae, strain Wijmberg), Bataviae (serogroup Bataviae, strain Pavia), Australis (serogroup Australis, strain Ballico), Zanoni (serogroup Pyrogenes, strain Zanoni), Saxkoebing (serogroup Sejroe, strain Mus 24), Sejroe (serogroup Sejroe, strain Topo 1), Poi (serogroup Javanica, strain Poi), Mini (serogroup Mini, strain Sari), Lora (serogroup Australis, strain Riccio 37), Hardjo (serogroup Sejroe, strain Farina), Autumnalis (serogroup Autumnalis, strain Akiyami A), Hebdomadis (serogroup Hebdomadis, strain Hebdomadis).
